# Some Integral Inequalities Involving Metrics

**DOI:** 10.3390/e23070871

**Published:** 2021-07-08

**Authors:** Ravi P. Agarwal, Mohamed Jleli, Bessem Samet

**Affiliations:** 1 Department of Mathematics, Texas A & M University-Kingsville, Kingsville, TX 78363, USA; Ravi.Agarwal@tamuk.edu; 2 Department of Mathematics, College of Science, King Saud University, P.O. Box 2455, Riyadh 11451, Saudi Arabia; jleli@ksu.edu.sa

**Keywords:** integral inequalities, metric, partial metric, sub-additive, convex, log-convex, σ-Lipschitzian

## Abstract

In this work, we establish some integral inequalities involving metrics. Moreover, some applications to partial metric spaces are given. Our results are extension of previous obtained metric inequalities in the discrete case.

## 1. Introduction

Metric inequalities provide powerful tools for the investigation of several problems from different branches of mathematics and sciences. In particular, from entropy and information theory (see e.g., [1,2,3]), fixed point theory (see e.g., [4,5,6,7,8]), geometry (see e.g., [9,10,11]) and telecommunication networks (see e.g., [12,13]).

In [14], Dragomir and Gosa established a polygonal type inequality and provided some applications to normed linear spaces and inner product spaces. We recall below the main result obtained in [14]. Let X be a nonempty set and ρ:X×X→[0,+∞) be a metric on X (see [15]), that is, for all a,b,c∈X,

- ρ(a,b)=0 if and only if a=b;
- ρ(a,b)=ρ(b,a);
- ρ(a,b)≤ρ(a,c)+ρ(c,b).

Let n≥2 be a natural number, ${\left\{x_{i}\right\}}_{i=1}^{n}\subset X$, and ${\left\{\mu_{i}\right\}}_{i=1}^{n}\subset \left[0,+\infty \right)$ with $\mu_{1}+\cdots \mu_{n}=1$. Then

(1) $$\sum_{1\leq i<j\leq n}\mu_{i}\mu_{j}\rho \left(x_{i},x_{j}\right)\leq \underset{x\in X}{\inf}\sum_{i=1}^{n}\mu_{i}\rho \left(x_{i},x\right).$$

It was shown also that (1) is sharp in the following sense: there exists n≥2 and ${\left\{\mu_{i}\right\}}_{i=1}^{n}\subset \left[0,+\infty \right)$ with $\mu_{1}+\cdots \mu_{n}=1$ such that, if

$$\sum_{1\leq i<j\leq n}\mu_{i}\mu_{j}\rho \left(x_{i},x_{j}\right)\leq c\underset{x\in X}{\inf}\sum_{i=1}^{n}\mu_{i}\rho \left(x_{i},x\right)$$

for some c>0, then c≥1. Inequality (1) can be interpreted as follows: If P is a polygon having *q* vertices and *M* is a point in the space, then the sum of all edges and diagonals of P is less than *q*-times the sum of the distances from *M* to the vertices of P.

Recently, inequality (1) has been extended by some authors. In [16], Karapinar and Noorwali derived a *b*-metric version of (1). In [17], Aydi and Samet proved (under the above assumptions) that

12∑1≤i<j≤nμiμj[ρ(xi,xj)]m≤infx∈X2∑i=1nμi[ρ(xi,x)]m+∑k=1m−1(mk)∑i=1nμi[ρ(xi,x)]k∑i=1nμi[ρ(xi,x)]m−k,

where m≥1 is a natural number. In [18] (see also [19]) Dragomir improved inequality (1) by proving that for all α>0,

(2) $$\sum_{1\leq i<j\leq n}\mu_{i}\mu_{j}{\left[\rho \left(x_{i},x_{j}\right)\right]}^{\alpha }\leq a_{\alpha }\underset{x\in X}{\inf}\sum_{i=1}^{n}\mu_{i}\left(1-\mu_{i}\right){\left[\rho \left(x_{i},x\right)\right]}^{\alpha },$$

where

(3) $$a_{\alpha }=\left\{\begin{array}{ccc} 2^{\alpha -1} & \mathrm{if} & \alpha \geq 1,\\ 1 & \mathrm{if} & 0<\alpha <1. \end{array}\right.$$

In this paper, our goal is to derive continuous versions of inequality (2). In the next section, we recall some basic definitions. In Section 3, we present and prove our obtained results. Finally, in Section 4, some applications to partial metric spaces are provided.

## 2. Some Definitions

Let ω:[0,+∞)→[0,+∞) be a given function. We say that ω is sub-additive, if

ω(y+z)≤ω(y)+ω(z),for all y,z≥0.

Some examples of sub-additive functions are given below:

- $\omega_{\alpha }\left(y\right)=y^{\alpha }$, y≥0, 0≤α≤1.
- ω(y)=|siny|, y≥0.
- ω(y)=arctany, y≥0.
- ω(y)=inf{y,1}, y≥0.
- $\omega \left(y\right)=\sqrt{1+y^{2}}$, y≥0.
- ω(y)=exp(−y), y≥0.

We say that ω is convex, if

ω(ϕy+(1−ϕ)z)≤ϕω(y)+(1−ϕ)ω(z),

for all 0<ϕ<1 and y,z≥0.

We say that ω is log-convex, if

$$\omega \left(\phi y+\left(1-\phi \right)z\right)\leq {\left[\omega \left(y\right)\right]}^{\phi }{\left[\omega \left(z\right)\right]}^{1-\phi },$$

for all 0<ϕ<1 and y,z≥0. Notice that, if ω is log-convex, then it is convex, but the converse is not true in general (see [20]).

We say that ω is σ-Lipschitzian, σ>0, if

|ω(y)−ω(z)|≤σ|y−z|,for all y,z≥0.

## 3. Results and Proofs

Let (X,ρ) be a metric space. Let x:[0,A]→X, A>0, be a continuous mapping. Let μ:[0,A]→[0,+∞) be a function satisfying the following conditions:

(H1) μ is continuous.
(H2) $\int_{0}^{A}\mu \left(s\right)\;ds=1$.

### 3.1. The Case: ω Is Sub-Additive

**Theorem** **1.** *Let ω:[0,+∞)→[0,+∞) be a continuous, nondecreasing, and sub-additive function. Then*

(4) $$\int_{0}^{A}\mu \left(t\right)\left(\int_{0}^{t}\mu \left(s\right)\omega \left(\rho (x(t),x(s))\right)\;ds\right)\;dt\leq \underset{u\in X}{\inf}\int_{0}^{A}\mu \left(s\right)\omega \left(\rho (x(s),u)\right)\;ds.$$

**Proof.**  Let u∈X be fixed. Then, for every t,s∈[0,A],

ρ(x(t),x(s))≤ρ(x(t),u)+ρ(u,x(s)).

Since ω is nondecreasing, the above inequality leads to

$$\omega \left(\rho (x(t),x(s))\right)\leq \omega \left(\rho (x(t),u)+\rho (u,x(s))\right).$$

Due to the sub-additivity of ω, it holds that

$$\omega \left(\rho (x(t),x(s))\right)\leq \omega \left(\rho (x(t),u)\right)+\omega \left(\rho (u,x(s))\right).$$

Multiplying the above inequality by μ(t)μ(s) (notice that μ≥0) and integrating over [0,A]×[0,A], we obtain

(5) $$\begin{array}{ccc} \int_{0}^{A}\int_{0}^{A}\mu \left(t\right)\mu \left(s\right)\omega \left(\rho (x(t),x(s))\right)\;dt\;ds & \leq & \int_{0}^{A}\int_{0}^{A}\mu \left(t\right)\mu \left(s\right)\omega \left(\rho (x(t),u)\right)\;dt\;ds\\ & & +\int_{0}^{A}\int_{0}^{A}\mu \left(t\right)\mu \left(s\right)\omega \left(\rho (u,x(s)\right)\;dt\;ds. \end{array}$$

On the other hand, by (H2), we have

$$\begin{array}{ccc} \int_{0}^{A}\int_{0}^{A}\mu \left(t\right)\mu \left(s\right)\omega \left(\rho (x(t),u)\right)\;dt\;ds & = & \left(\int_{0}^{A}\mu \left(s\right)\;ds\right)\left(\int_{0}^{A}\mu \left(t\right)\omega \left(\rho (x(t),u)\right)\;dt\right)\\ & = & \int_{0}^{A}\mu \left(t\right)\omega \left(\rho (x(t),u)\right)\;dt. \end{array}$$

Similarly,

$$\int_{0}^{A}\int_{0}^{A}\mu \left(t\right)\mu \left(s\right)\omega \left(\rho (u,x(s))\right)\;dt\;ds=\int_{0}^{A}\mu \left(s\right)\omega \left(\rho (u,x(s))\right)\;ds.$$

Combining the above inequalities, we obtain

(6) $$\begin{array}{cc} \; & \int_{0}^{A}\int_{0}^{A}\mu \left(t\right)\mu \left(s\right)\omega \left(\rho (x(t),u)\right)\;dt\;ds+\int_{0}^{A}\int_{0}^{A}\mu \left(t\right)\mu \left(s\right)\omega \left(\rho (u,x(s))\right)\;dt\;ds\\ \; & =2\int_{0}^{A}\mu \left(s\right)\omega \left(\rho (u,x(s))\right)\;ds. \end{array}$$

Moreover, we have

(7) $$\begin{array}{ccc} \int_{0}^{A}\int_{0}^{A}\mu \left(t\right)\mu \left(s\right)\omega \left(\rho (x(t),x(s))\right)\;dt\;ds & = & \int_{0}^{A}\mu \left(t\right)\left(\int_{0}^{t}\mu \left(s\right)\omega \left(\rho (x(t),x(s))\right)\;ds\right)\;dt\\ & & +\int_{0}^{A}\mu \left(t\right)\left(\int_{t}^{A}\mu \left(s\right)\omega \left(\rho (x(t),x(s))\right)\;ds\right)\;dt. \end{array}$$

On the other hand, using Fubini’s theorem and the symmetry of ρ, we obtain

(8) $$\int_{0}^{A}\mu \left(t\right)\left(\int_{t}^{A}\mu \left(s\right)\omega \left(\rho (x(t),x(s))\right)\;ds\right)\;dt=\int_{0}^{A}\mu \left(t\right)\left(\int_{0}^{t}\mu \left(s\right)\omega \left(\rho (x(t),x(s))\right)\;ds\right)\;dt.$$

Therefore, by (7) and (8), we deduce that

(9) $$\int_{0}^{A}\int_{0}^{A}\mu \left(t\right)\mu \left(s\right)\omega \left(\rho (x(t),x(s))\right)\;dt\;ds=2\int_{0}^{A}\mu \left(t\right)\left(\int_{0}^{t}\mu \left(s\right)\omega \left(\rho (x(t),x(s))\right)\;ds\right)\;dt.$$

Finally, (4) follows from (5), (6) and (9). □

Next, we study some special cases of ω. In the case $\omega \left(y\right)=y^{\alpha }$, 0<α≤1, we deduce from Theorem 1 the

**Corollary** **1.** *Let 0<α≤1. Then*

$$\int_{0}^{A}\mu \left(t\right)\left(\int_{0}^{t}\mu \left(s\right){\left[\rho (x(t),x(s))\right]}^{\alpha }\;ds\right)\;dt\leq \underset{u\in X}{\inf}\int_{0}^{A}\mu \left(s\right){\left[\rho (x(s),u)\right]}^{\alpha }\;ds.$$

In the special case when $\omega \left(y\right)=\sqrt{\alpha +y^{2}}$, α>0, we deduce from Theorem 1 the

**Corollary** **2.** *Let α>0. Then*

$$\int_{0}^{A}\mu \left(t\right)\left(\int_{0}^{t}\mu \left(s\right)\sqrt{\alpha +{\left[\rho \left(x\left(t\right),x\left(s\right)\right)\right]}^{2}}\;ds\right)\;dt\leq \underset{u\in X}{\inf}\int_{0}^{A}\mu \left(s\right)\sqrt{\alpha +{\left[\rho \left(x\left(s\right),u\right)\right]}^{2}}\;ds.$$

In the case when ω(y)=arctan y, we deduce from Theorem 1 the

**Corollary** **3.** *The following inequality holds:*

$$\int_{0}^{A}\mu \left(t\right)\left(\int_{0}^{t}\mu \left(s\right)\arctan\left(\rho (x(t),x(s))\right)\;ds\right)\;dt\leq \underset{u\in X}{\inf}\int_{0}^{A}\mu \left(s\right)\arctan\left(\rho (x(s),u)\right)\;ds.$$

### 3.2. The Case: ω Is Convex

**Theorem** **2.** *Let ω:[0,+∞)→[0,+∞) be a nondecreasing and convex function. Then*

(10) $$\int_{0}^{A}\mu \left(t\right)\left(\int_{0}^{t}\mu \left(s\right)\omega \left(\frac{\rho (x(t),x(s))}{2}\right)\;ds\right)\;dt\leq \frac{1}{2}\underset{u\in X}{\inf}\int_{0}^{A}\mu \left(s\right)\omega \left(\rho (x(s),u)\right)\;ds.$$

**Proof.**  Fix u∈X. Since ω is nondecreasing, we have

$$\omega \left(\frac{\rho (x(t),x(s))}{2}\right)\leq \omega \left(\frac{\rho (x(t),u)+\rho (u,x(s))}{2}\right).$$

Due to the convexity of ω, the above inequality leads to

$$\omega \left(\frac{\rho (x(t),x(s))}{2}\right)\leq \frac{1}{2}\left[\omega \left(\rho (x(t),u)\right)+\omega \left(\rho (u,x(s))\right)\right].$$

Multiplying the above inequality by μ(t)μ(s) and integrating over [0,A]×[0,A], we obtain

(11) $$\begin{array}{ccc} \int_{0}^{A}\int_{0}^{A}\mu \left(t\right)\mu \left(s\right)\omega \left(\frac{\rho (x(t),x(s))}{2}\right)\;dt\;ds & \leq & \frac{1}{2}\int_{0}^{A}\int_{0}^{A}\mu \left(t\right)\mu \left(s\right)\omega \left(\rho (x(t),u)\right)\;dt\;ds\\ & & +\frac{1}{2}\int_{0}^{A}\int_{0}^{A}\mu \left(t\right)\mu \left(s\right)\omega \left(\rho (u,x(s)\right)\;dt\;ds. \end{array}$$

Proceeding as in the proof of Theorem 1, we obtain

(12) $$\int_{0}^{A}\int_{0}^{A}\mu \left(t\right)\mu \left(s\right)\omega \left(\frac{\rho (x(t),x(s))}{2}\right)\;dt\;ds=2\int_{0}^{A}\mu \left(t\right)\left(\int_{0}^{t}\mu \left(s\right)\omega \left(\frac{\rho (x(t),x(s))}{2}\right)\;ds\right)\;dt$$

and

(13) $$\begin{array}{cc} \; & \frac{1}{2}\int_{0}^{A}\int_{0}^{A}\mu \left(t\right)\mu \left(s\right)\omega \left(\rho (x(t),u)\right)\;dt\;ds+\frac{1}{2}\int_{0}^{A}\int_{0}^{A}\mu \left(t\right)\mu \left(s\right)\omega \left(\rho (u,x(s)\right)\;dt\;ds\\ \; & =\int_{0}^{A}\mu \left(s\right)\omega \left(\rho (x(s),u)\right)\;ds. \end{array}$$

Finally, combining (11), (12), and (13), (10) follows. □

In the special case when $\omega \left(y\right)=y^{\alpha }$, α>1, by Theorem 2, we deduce the following result.

**Corollary** **4.** *Let α>1. Then*

$$\int_{0}^{A}\mu \left(t\right)\left(\int_{0}^{t}\mu \left(s\right){\left[\rho (x(t),x(s))\right]}^{\alpha }\;ds\right)\;dt\leq 2^{\alpha -1}\underset{u\in X}{\inf}\int_{0}^{A}\mu \left(s\right){\left[\rho (x(s),u)\right]}^{\alpha }\;ds.$$

### 3.3. The Case: ω Is log-Convex

**Theorem** **3.** *Let ω:[0,+∞)→[0,+∞) be a nondecreasing and log-convex function. Then*

(14) $$\int_{0}^{A}\mu \left(t\right)\left(\int_{0}^{t}\mu \left(s\right)\omega \left(\frac{\rho (x(t),x(s))}{2}\right)\;ds\right)\;dt\leq \frac{1}{2}\underset{u\in X}{\inf}{\left(\int_{0}^{A}\mu \left(s\right)\sqrt{\omega \left(\rho (x(s),u)\right)}\;ds\right)}^{2}.$$

**Proof.**  Fix u∈X. Since ω is nondecreasing, we have

$$\omega \left(\frac{\rho (x(t),x(s))}{2}\right)\leq \omega \left(\frac{\rho (x(t),u)+\rho (u,x(s))}{2}\right).$$

Due to the log-convexity of ω, the above inequality leads to

$$\omega \left(\frac{\rho (x(t),x(s))}{2}\right)\leq \sqrt{\omega \left(\rho (x(t),u)\right)}\sqrt{\omega \left(\rho (u,x(s))\right)}.$$

Multiplying the above inequality by μ(t)μ(s) and integrating over [0,A]×[0,A], we obtain

(15) $$\begin{array}{cc} \; & \int_{0}^{A}\int_{0}^{A}\mu \left(t\right)\mu \left(s\right)\omega \left(\frac{\rho (x(t),x(s))}{2}\right)\;dt\;ds\\ \; & \leq \int_{0}^{A}\int_{0}^{A}\mu \left(t\right)\mu \left(s\right)\sqrt{\omega \left(\rho (x(t),u)\right)}\sqrt{\omega \left(\rho (u,x(s))\right)}\;dt\;ds. \end{array}$$

On the other hand,

(16) $$\int_{0}^{A}\int_{0}^{A}\mu \left(t\right)\mu \left(s\right)\sqrt{\omega \left(\rho (x(t),u)\right)}\sqrt{\omega \left(\rho (u,x(s))\right)}\;dt\;ds={\left(\int_{0}^{A}\mu \left(s\right)\sqrt{\omega \left(\rho (x(s),u)\right)}\;ds\right)}^{2}.$$

Hence, using (12), (15) and (16), (14) follows. □

Consider the special case $\omega \left(y\right)=\exp\left(y^{\alpha }\right)$, α≥1. By Theorem 3, we obtain the

**Corollary** **5.** *Let α≥1. Then*

$$\int_{0}^{A}\mu \left(t\right)\left(\int_{0}^{t}\mu \left(s\right)\exp\left[{\left(\frac{\rho (x(t),x(s))}{2}\right)}^{\alpha }\right]\;ds\right)\;dt\leq \frac{1}{2}\underset{u\in X}{\inf}{\left(\int_{0}^{A}\mu \left(s\right)\sqrt{\exp\left[{\left(\rho (x(s),u)\right)}^{\alpha }\right]}\;ds\right)}^{2}.$$

### 3.4. The Case: ω Is σ-Lipschitzian

**Theorem** **4.** *Let ω:[0,+∞)→[0,+∞) be a nondecreasing and σ-Lipschitzian function, σ>0. Then*

(17) $$\int_{0}^{A}\mu \left(t\right)\left(\int_{0}^{t}\mu \left(s\right)\omega \left(\rho (x(t),x(s))\right)\;ds\right)\;dt\leq \sigma \underset{u\in X}{\inf}\left[\int_{0}^{A}\mu \left(s\right)\omega \left(\rho (x(s),u)\right)\;ds\right]+\frac{\omega (0)}{2}.$$

**Proof.**  Let u∈X be fixed. Then, for every t,s∈[0,A],

ρ(x(t),x(s))≤ρ(x(t),u)+ρ(u,x(s)).

Since ω is nondecreasing, the above inequality leads to

$$\omega \left(\rho (x(t),x(s))\right)\leq \omega \left(\rho (x(t),u)+\rho (u,x(s))\right).$$

On the other hand, since ω is σ-Lipschitzian, we have

$$\begin{array}{ccc} \omega \left(\rho (x(t),u)+\rho (u,x(s))\right) & = & \left[\omega \left(\rho (x(t),u)+\rho (u,x(s))\right)-\omega \left(0\right)\right]+\omega \left(0\right)\\ & \leq & \sigma \left[\rho (x(t),u)+\rho (u,x(s))\right]+\omega \left(0\right). \end{array}$$

Hence, it holds that

$$\omega \left(\rho (x(t),x(s))\right)\leq \sigma \left[\rho (x(t),u)+\rho (u,x(s))\right]+\omega \left(0\right).$$

Multiplying the above inequality by μ(t)μ(s) and integrating over [0,A]×[0,A], we obtain

(18) $$\begin{array}{cc} & \int_{0}^{A}\int_{0}^{A}\mu \left(t\right)\mu \left(s\right)\omega \left(\rho (x(t),x(s))\right)\;dt\;ds\\ & \leq \sigma \int_{0}^{A}\int_{0}^{A}\mu \left(t\right)\mu \left(s\right)\omega \left(\rho (x(t),u)\right)\;dt\;ds+\sigma \int_{0}^{A}\int_{0}^{A}\mu \left(t\right)\mu \left(s\right)\omega \left(\rho (u,x(s)\right)\;dt\;ds\\ & +\omega \left(0\right)\int_{0}^{A}\int_{0}^{A}\mu \left(t\right)\mu \left(s\right)\;dt\;ds\\ & =\sigma \int_{0}^{A}\int_{0}^{A}\mu \left(t\right)\mu \left(s\right)\omega \left(\rho (x(t),u)\right)\;dt\;ds+\sigma \int_{0}^{A}\int_{0}^{A}\mu \left(t\right)\mu \left(s\right)\omega \left(\rho (u,x(s)\right)\;dt\;ds+\omega \left(0\right). \end{array}$$

Next, using (6), (9) and (18), we deduce that

$$2\int_{0}^{A}\mu \left(t\right)\left(\int_{0}^{t}\mu \left(s\right)\omega \left(\rho (x(t),x(s))\right)\;ds\right)\;dt\leq 2\sigma \int_{0}^{A}\mu \left(s\right)\omega \left(\rho (u,x(s))\right)\;ds+\omega \left(0\right),$$

which yields (17). □

### 3.5. The Case: $\mu \equiv A^{-1}$

Consider now the case when

$$\mu \left(t\right)=A^{-1},\;\mathrm{for}\;\mathrm{all}\;t\in \left[0,A\right].$$

Then by Theorems 1–4, we deduce the following inequalities.

**Corollary** **6.** *Let ω:[0,+∞)→[0,+∞) be a continuous, nondecreasing, and sub-additive function. Then*

$$\int_{0}^{A}\left(\int_{0}^{t}\omega \left(\rho (x(t),x(s))\right)\;ds\right)\;dt\leq A\underset{u\in X}{\inf}\int_{0}^{A}\omega \left(\rho (x(s),u)\right)\;ds.$$

**Corollary** **7.** *Let ω:[0,+∞)→[0,+∞) be a nondecreasing and convex function. Then*

$$\int_{0}^{A}\left(\int_{0}^{t}\omega \left(\frac{\rho (x(t),x(s))}{2}\right)\;ds\right)\;dt\leq \frac{A}{2}\underset{u\in X}{\inf}\int_{0}^{A}\omega \left(\rho (x(s),u)\right)\;ds.$$

**Corollary** **8.** *Let α>0. Then*

$$\int_{0}^{A}\left(\int_{0}^{t}{\left[\rho (x(t),x(s))\right]}^{\alpha }\;ds\right)\;dt\leq a_{\alpha }A\underset{u\in X}{\inf}\int_{0}^{A}{\left[\rho (x(s),u)\right]}^{\alpha }\;ds,$$

*where $a_{\alpha }$ is given by (3).*

**Corollary** **9.** *Let ω:[0,+∞)→[0,+∞) be a nondecreasing and log-convex function. Then*

$$\int_{0}^{A}\left(\int_{0}^{t}\omega \left(\frac{\rho (x(t),x(s))}{2}\right)\;ds\right)\;dt\leq \frac{1}{2}\underset{u\in X}{\inf}{\left(\int_{0}^{A}\sqrt{\omega \left(\rho (x(s),u)\right)}\;ds\right)}^{2}.$$

**Corollary** **10.** *Let ω:[0,+∞)→[0,+∞) be a nondecreasing and σ-Lipschitzian function, σ>0. Then*

$$\int_{0}^{A}\left(\int_{0}^{t}\omega \left(\rho (x(t),x(s))\right)\;ds\right)\;dt\leq \sigma A\underset{u\in X}{\inf}\left[\int_{0}^{A}\omega \left(\rho (x(s),u)\right)\;ds\right]+\frac{A^{2}\omega \left(0\right)}{2}.$$

## 4. Applications to Partial Metrics

Let X be a nonempty set and ξ:X×X→[0,+∞) be a partial metric on X (see e.g., [21]), i.e., for all x,y,z∈X,

- ξ(x,y)=ξ(x,x)=ξ(y,y)=0 if and only if x=y;
- ξ(x,x)≤ξ(x,y);
- ξ(x,y)=ξ(y,x);
- ξ(x,y)≤ξ(x,z)+ξ(z,y)−ξ(z,z).

Let x:[0,A]→X, A>0, be a continuous mapping. Let μ:[0,A]→[0,+∞) be a function satisfying (H1) and (H2).

**Corollary** **11.** *Let ω:[0,+∞)→[0,+∞) be a continuous, nondecreasing, and sub-additive function. Then*

(19) $$\begin{array}{cc} \; & \int_{0}^{A}\mu \left(t\right)\left(\int_{0}^{t}\mu \left(s\right)\omega \left(2\xi (x(t),x(s))-\xi (x(t),x(t))-\xi (x(s),x(s))\right)\;ds\right)\;dt\\ \; & \leq \underset{u\in X}{\inf}\int_{0}^{A}\mu \left(s\right)\omega \left(2\xi (x(s),u)-\xi (x(s),x(s))-\xi (u,u)\right)\;ds. \end{array}$$

**Proof.**  Observe that the mapping ρ:X×X→[0,+∞) defined by

ρ(u,v)=2ξ(u,v)−ξ(u,u)−ξ(v,v),(u,v)∈X×X,

is a metric on X. Hence, applying Theorem 1 with ρ defined as above, (19) follows. □

Similarly, by Theorems 2–4, we obtain the following inequalities.

**Corollary** **12.** *Let ω:[0,+∞)→[0,+∞) be a nondecreasing and convex function. Then*

$$\begin{array}{cc} & \int_{0}^{A}\mu \left(t\right)\left(\int_{0}^{t}\mu \left(s\right)\omega \left(\frac{2\xi (x(t),x(s))-\xi (x(t),x(t))-\xi (x(s),x(s))}{2}\right)\;ds\right)\;dt\\ & \leq \frac{1}{2}\underset{u\in X}{\inf}\int_{0}^{A}\mu \left(s\right)\omega \left(2\xi (x(s),u)-\xi (x(s),x(s))-\xi (u,u)\right)\;ds. \end{array}$$

**Corollary** **13.** *Let ω:[0,+∞)→[0,+∞) be a nondecreasing and log-convex function. Then*

$$\begin{array}{cc} & \int_{0}^{A}\mu \left(t\right)\left(\int_{0}^{t}\mu \left(s\right)\omega \left(\frac{2\xi (x(t),x(s))-\xi (x(t),x(t))-\xi (x(s),x(s))}{2}\right)\;ds\right)\;dt\\ & \leq \frac{1}{2}\underset{u\in X}{\inf}{\left(\int_{0}^{A}\mu \left(s\right)\sqrt{\omega \left(2\xi (x(s),u)-\xi (x(s),x(s))-\xi (u,u)\right)}\;ds\right)}^{2}. \end{array}$$

**Corollary** **14.** *Let ω:[0,+∞)→[0,+∞) be a nondecreasing and σ-Lipschitzian function, σ>0. Then*

$$\begin{array}{cc} & \int_{0}^{A}\mu \left(t\right)\left(\int_{0}^{t}\mu \left(s\right)\omega \left(2\xi (x(t),x(s))-\xi (x(t),x(t))-\xi (x(s),x(s))\right)\;ds\right)\;dt\\ & \leq \sigma \underset{u\in X}{\inf}\left[\int_{0}^{A}\mu \left(s\right)\omega \left(2\xi (x(s),u)-\xi (x(s),x(s))-\xi (u,u)\right)\;ds\right]+\frac{\omega (0)}{2}. \end{array}$$

## 5. Conclusions

Metric inequalities provide powerful tools for the study of several problems from different branches of mathematics and sciences. New integral inequalities involving metrics, sub-additive, convex, log-convex, and σ-Lipschitzian functions are established in this work, and some applications to partial metric spaces are provided. The obtained results are continuous versions of some discrete metric inequalities obtained in [14,18,19].

## Data Availability

Not applicable.
